# lncRNA H19/miR-675 axis regulates cardiomyocyte apoptosis by targeting VDAC1 in diabetic cardiomyopathy

**DOI:** 10.1038/srep36340

**Published:** 2016-10-31

**Authors:** Xiangquan Li, Hao Wang, Biao Yao, Weiting Xu, Jianchang Chen, Xiang Zhou

**Affiliations:** 1 Department of Cardiology, The First People’s Hospital of Kunshan Affiliated to Jiangsu University, Kunshan, China; 2 Department of Cardiology, The First Affiliated Hospital of Nanjing Medical University, Nanjing, China; 3 Department of Cardiology, The Second Affiliated Hospital of Soochow University, Suzhou, China

## Abstract

We previously established a rat model of diabetic cardiomyopathy (DCM) and found that the expression of lncRNA H19 was significantly downregulated. The present study was designed to investigate the pathogenic role of H19 in the development of DCM. Overexpression of H19 in diabetic rats attenuated oxidative stress, inflammation and apoptosis, and consequently improved left ventricular function. High glucose was associated with reduced H19 expression and increased cardiomyocyte apoptosis. To explore the molecular mechanisms involved, we performed *in vitro* experiments using cultured neonatal rat cardiomyocytes. Our results showed that miR-675 expression was decreased in cardiomyocytes transfected with H19 siRNA. The 3′UTR of VDAC1 was cloned downstream of a luciferase reporter construct and cotransfected into HEK293 cells with miR-675 mimic. The results of luciferase assay indicated that VDAC1 might be a direct target of miR-675. The expression of VDAC1 was upregulated in cardiomyocytes transfected with miR-675 antagomir, which consequently promotes cellular apoptosis. Moreover, enforced expression of H19 was found to reduce VDAC1 expression and inhibit apoptosis in cardiomyocytes exposed to high glucose. In conclusion, our study demonstrates that H19/miR-675 axis is involved in the regulation of high glucose-induced apoptosis by targeting VDAC1, which may provide a novel therapeutic strategy for the treatment of DCM.

Diabetic cardiomyopathy (DCM), which was first described by Rubler *et al*.^1^ in 1972, is defined as myocardial dysfunction occurring in patients with diabetes in the absence of coronary artery disease, hypertension, or valvular heart disease^2^. Increasing evidence has demonstrated that inflammation, oxidative stress, mitochondrial dysfunction, impaired calcium handling, renin-angiotensin system activation, cardiomyocyte apoptosis are potentially involved in the pathogenesis of DCM^3^. To further explore the molecular mechanisms of DCM, we established a rat model of DCM and carried out a microarray to identify the differentially expressed long non-coding RNAs (lncRNAs) in myocardial tissue. Our findings showed that the expression of lncRNA H19 was significantly downregulated in diabetic rats.

The H19 gene belongs to a highly conserved imprinted gene cluster and plays crucial roles in embryonal development and growth control^4^. H19 expression is strongly induced during embryogenesis and downregulated after birth, except in adult skeletal muscle and heart. Recently, it has been reported that H19 acts as the precursor of miR-675 and the latter mediates the function of H19 in several biological processes. H19 exon1 encodes for two conserved miRNAs, miR675-3p and miR675-5p, and H19/miR-675 axis is critically involved in carcinogenesis, progression and metastasis of several types of cancers^5^,^6^,^7^,^8^. In the present study, we aimed to investigate the pathophysiological role of H19/miR-675 axis in the development of DCM. Using *in silico* prediction and *in vitro* functional assays, we confirmed that voltage-dependent anion channel 1 (VDAC1) is a direct target of miR-675. VDAC1 is recognized as a critical protein required for the mitochondria-mediated apoptosis which is an important molecular mechanism responsible for impaired cardiac function associated with diabetes. Our findings demonstrate that H19-derived miR-675, through downregulation of its target gene VDAC1, regulates cardiomyocyte apoptosis in the pathogenesis of DCM.

## Methods

### Animal model and treatment

All experiments and procedures were approved by the Animal Ethics Committee of Soochow University and were carried out in accordance with the Guide for the Care and Use of Laboratory Animals. Male Sprague–Dawley rats weighing 200–250 g were purchased from the Experimental Animal Center of Soochow University. The diabetic rat model was established by a single intraperitoneal injection of streptozotocin (65 mg/kg, Sigma) as previously described^9^. The tail vein blood glucose levels were measured one week after streptozotocin injection using a glucometer (Accu-Chek, Roche Diagnostics). Only rats with blood glucose levels ≥16.7 mmol/l were considered diabetic in this study. Diabetic rats were intracoronary injected with lentivirus of pcDNA-H19 (DM + pcDNA-H19) or empty vector (DM + pcDNA vector), and were then kept for 12 weeks together with the normal rats (Control) and the diabetic rats without lentivirus injection (DM).

### Cardiomyocyte culture

The hearts were surgically removed from 1–2 days old rats and washed instantly in cold D-Hanks solution. The ventricular tissues were cut into small pieces and then underwent a series of digestion at 37 °C in D-Hanks solution containing 1.2 mg/mL pancreatin and 0.14 mg/mL collagenase (Gibco). After centrifugation, the cells were suspended in Dulbecco’s modified Eagle’s medium (Gibco) containing 20% calf serum, 100 U/ml penicillin and 100 mg/ml streptomycin. The dissociated cells were preplated at 37 °C for 1 hour to separate cardiomyocytes by adherence of cardiac fibroblasts. Thereafter, the cells were collected and diluted to 1 × 10^6^ cells/ml and plated in 1% gelatin-coated different culture dishes. Cardiomyocytes were incubated at 37 °C and 5% CO_2_ in a humidified incubator.

### Echocardiographic study

Cardiac structure and function were analyzed by echocardiography performed as previously described^10^. Left ventricular end-diastolic diameter (LVEDD) and left ventricular end-systolic diameter (LVESD) were measured from the parasternal long-axis view. Left ventricular fractional shortening (LVFS) and left ventricular ejection fraction (LVEF) were determined to evaluate left ventricular systolic function. The mitral peak flow velocities at early diastole (E) and atrial contraction (A) were detected by pulsed Doppler technique. Tissue Doppler imaging was obtained from the lateral mitral valve annulus, and early diastolic (Ea) and late diastolic (Aa) mitral annular velocities were measured. The E/A and Ea/Aa ratios were calculated and used as indices of left ventricular diastolic function. All measurements in this study were averaged for 3 consecutive cardiac cycles.

### Inflammatory cytokines detection

The concentrations of inflammatory markers in myocardial tissues, including tumor necrosis factor-α (TNF-α), interleukin-1β (IL-1β), and interleukin-6 (IL-6), were measured using commercial enzyme-linked immunosorbent assay (ELISA) kits (R&D Systems) according to the manufacturer’s protocol.

### Oxidative stress measurement

Oxidative stress was assessed by detecting malondialdehyde (MDA), superoxide dismutase (SOD) and glutathione peroxidase (GSH-Px) in myocardial tissues according to the instructions of detection kits (Jiancheng Biotech).

### TUNEL staining

Cellular apoptosis was determined using the terminal deoxynucleotidyl transferase-mediated dUTP nick-end labeling (TUNEL) assay. The apoptotic index was calculated as the percentage of TUNEL-positive cells divided by the total number of cells. TUNEL *in situ* cell death detection kits were purchased from Promega.

### Annexin V-FITC/PI staining

Cardiomyocytes were stained with Annexin V-FITC and propidium iodide (PI) and then subjected to flow cytometry to detect apoptosis. The apoptotic rate was calculated as the percentage of Annexin V-positive and PI-negative cells divided by the total number of cells in the gated region. The Annexin V-FITC apoptosis detection kit was obtained from BD Pharmingen.

### Luciferase reporter assay

To confirm whether VDAC1 was a direct target of miR-675, we performed luciferase reporter experiments in HEK293 cells. The 3′-untranslated regions (UTR) of VDAC1 was cloned into the downstream of luciferase gene to generate Luc-VDAC1-Wt vector. The 3′-UTR without predicted miR-675 binding site was constructed to generate Luc-VDAC1-Mut vector. For luciferase assay, cells were plated in 24-well culture plates, and then transfected with either wild-type or mutant construct with and without miRNA mimic or negative control. Luciferase activity was detected 48 hours after transfection using the Dual Luciferase Reporter Assay System (Promega).

### Real-time PCR

Total RNA was isolated from myocardial tissues and neonatal cardiomyocytes using TRIzol Reagent (Invitrogen). RNA was reverse transcribed using SuperScript First Strand cDNA System (Invitrogen) according to the manufacturer’s instructions. Quantitative PCR was conducted using SYBR Green Taq ReadyMix (Sigma) on an Applied Biosystems 7500 Real-Time PCR System, and GAPDH was used as the endogenous control. The primer sequences are as follows: H19, 5′-TATCGGACTCCAGAGGGATT-3′ and 5′-GGCATACAGTGCACCAAGTC-3′; VDAC1, 5′-TGCCATTTTAGGGTGGAGAG-3′ and 5′-GTGCGGCTACAAGAGGAGTC-3′. For miRNA real-time PCR, the miR-specific primers from the TaqMan miR assays (Applied Biosystems) were applied, and the snRNA U6 was used as an endogenous reference.

### Western blotting

Protein homogenates were prepared from cardiac tissues and neonatal cardiomyocytes. Equal amounts of protein (50 μg) were separated by SDS/PAGE (10% gel), transferred on to nitrocellulose membranes and blocked by 5% non-fat milk. The membranes were incubated with primary antibodies at 4 °C overnight, and then were incubated with horseradish peroxidase-conjugated secondary antibodies at room temperature for 1 hour. The antibodies were purchased from Cell Signaling Technology and were used at manufacturer-recommended dilutions. The immunocomplexes were visualized using an enhanced chemiluminescence detection kit (Amersham Biosciences).

### Statistical analysis

All data in this study are expressed as mean ± SD, and differences between groups were determined using analysis of variance (ANOVA) with SPSS version 18.0. The Bonferroni post-hoc test was used for multiple comparisons if the ANOVA was significant. *P* < 0.05 was considered statistically significant.

## Results

### H19 is involved in the regulation of cardiomyocyte apoptosis in DCM

**Figure 1:**
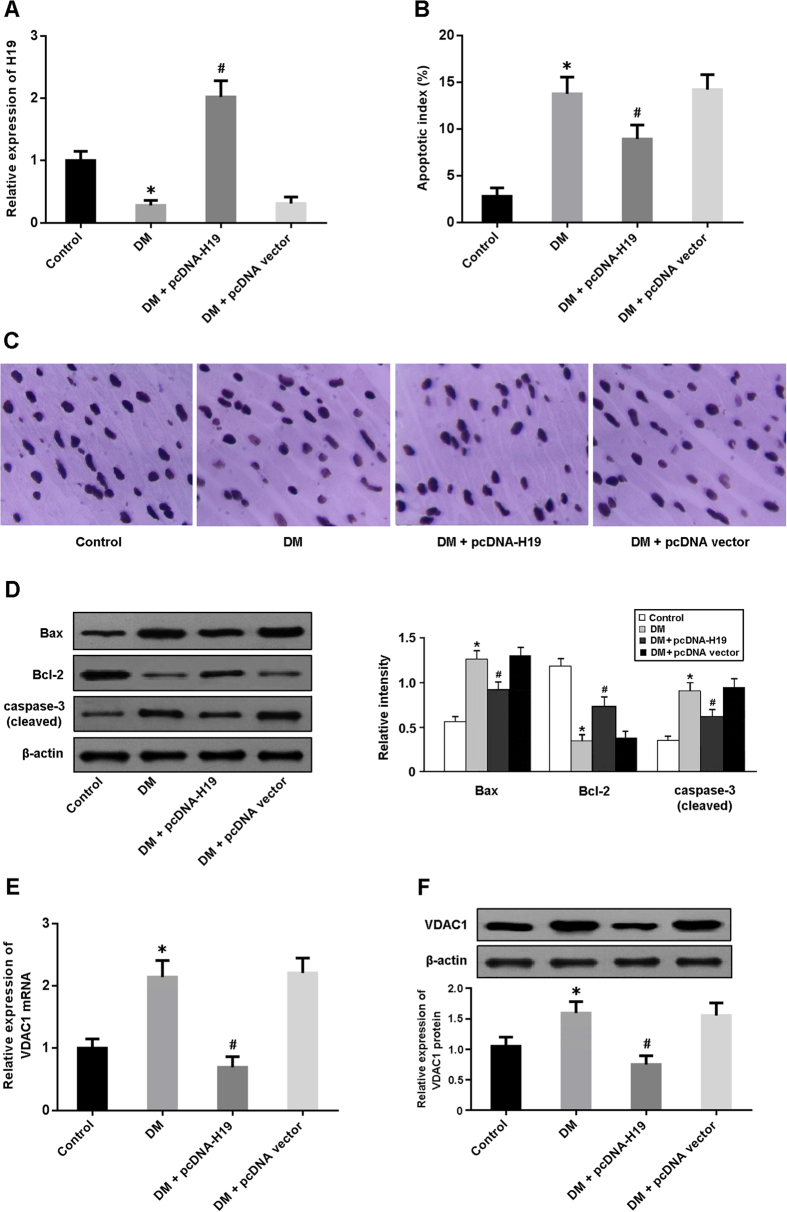
H19 is involved in the regulation of cardiomyocyte apoptosis in diabetic rats. (**A**) The expression of H19 in cardiac tissue was detected by real-time PCR. (**B**) Cardiomyocyte apoptosis was assessed by TUNEL staining and the apoptotic index was calculated. (**C**) Representative micrographs of myocardial tissue stained with TUNEL. (**D**) The expression of apoptosis-regulatory proteins (Bax, Bcl-2 and caspase-3) was determined by Western blot. (**E**,**F**) The mRNA and protein expression of VDAC1 was detected by real-time PCR and Western blot. *P < 0.05 compared with control; ^#^P < 0.05 compared with DM.

As shown in Fig. 1A, the expression of H19 was significantly downregulated in the myocardium of diabetic rats and upregulated after injection with lentivirus pcDNA-H19. Cardiomyocyte apoptosis was determined by TUNEL staining, and the apoptotic index was found to be higher in the DM group and lower in the DM + pcDNA-H19 group (Fig. 1B,C). The cleaved caspase-3 expression and Bax/Bcl-2 ratio were markedly increased in diabetic rats and decreased following treatment with pcDNA-H19 (Fig. 1D). In addition, the mRNA and protein expression of VDAC1 was elevated in diabetic rats and reduced after administration of lentivirus pcDNA-H19 (Fig. 1E,F).

### Enforced expression of H19 improves cardiac structure and function in DCM

**Figure 2:**
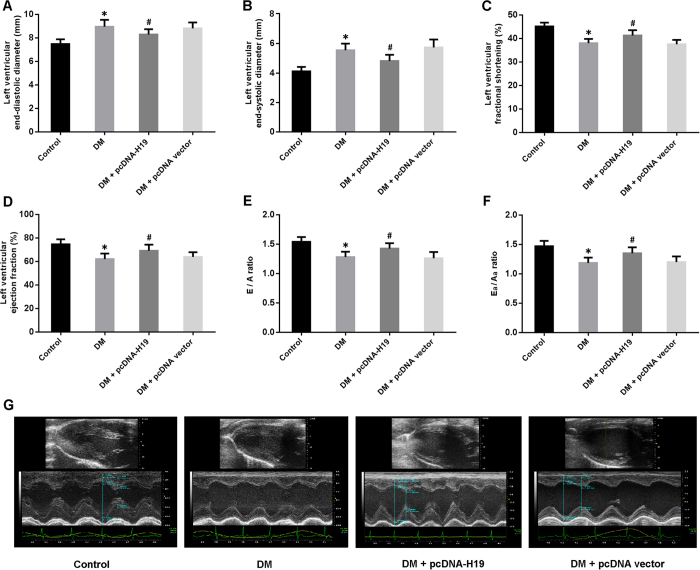
Enforced expression of H19 improves cardiac structure and function in diabetic rats. (**A**,**B**) Left ventricular structure was determined by echocardiography. (**C**–**F**) Left ventricular systolic and diastolic function was evaluated by echocardiography. (**G**) Representative echocardiographic images in measurement of cardiac structure. E: peak velocity of early ventricular filling; A: peak velocity of transmitral flow during atrial contraction; Ea: early diastolic mitral annular velocity; Aa: late diastolic mitral annular velocity. *P < 0.05 compared with control; ^#^P < 0.05 compared with DM.

The echocardiographic data are presented in Fig. 2. LVEDD and LVESD were remarkably increased in the DM group and decreased in the DM + pcDNA-H19 group (Fig. 2A,B,G). LVFS, LVEF, E/A and Ea/Aa ratios, indicators of left ventricular systolic and diastolic function, were found to be reduced in diabetic rats, whereas H19 overexpression could markedly improve hyperglycemia-induced left ventricular dysfunction (Fig. 2C–F).

### Overexpression of H19 alleviates inflammation and oxidative stress in DCM

**Figure 3:**
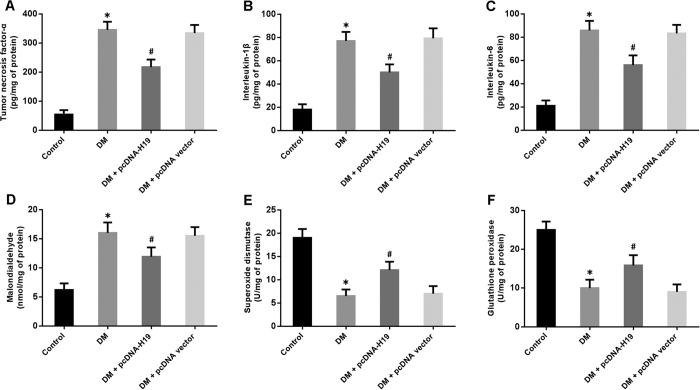
Overexpression of H19 attenuates inflammation and oxidative stress in diabetic rats. (**A**–**C**) The levels of inflammatory cytokines in cardiac tissue were measured by ELISA. (**D**–**F**) Oxidative stress was evaluated by detection of oxidation-related proteins in myocardial tissue. *P < 0.05 compared with control; ^#^P < 0.05 compared with DM.

The inflammatory markers were measured by ELISA and the results are shown in Fig. 3A–C. TNF-α, IL-1β and IL-6 levels were significantly elevated in the myocardium of diabetic rats, whereas H19 overexpression reduced concentrations of these inflammatory cytokines. In addition, oxidative stress was assessed by detecting MDA levels and SOD and GSH-Px activities. Our findings indicated that enforced expression of H19 could remarkably attenuate hyperglycemia-mediated oxidative stress in myocardial tissue (Fig. 3D–F).

### H19/miR-675 axis is involved in the regulation of high glucose-induced apoptosis

**Figure 4:**
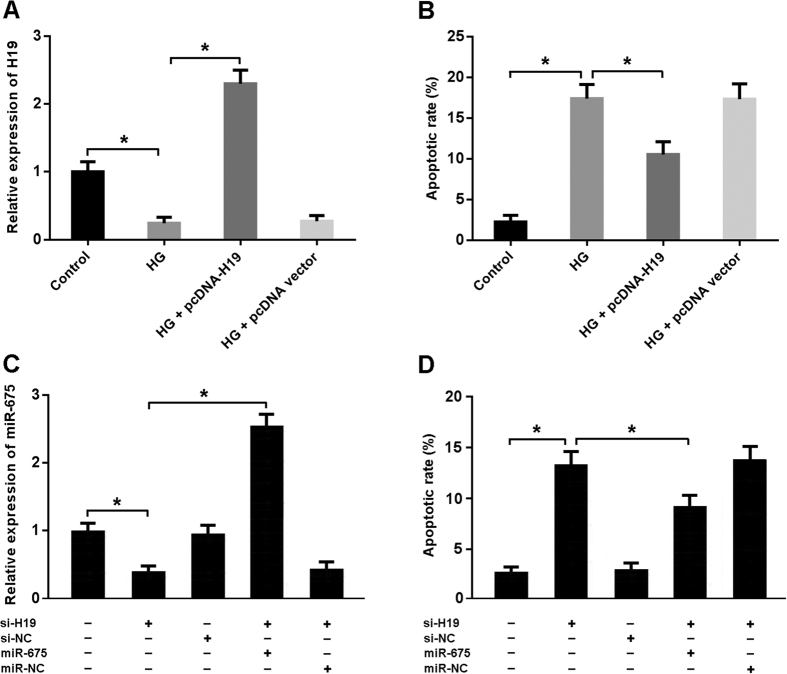
H19/miR-675 axis is involved in the regulation of high glucose-induced apoptosis. (**A**) Cardiomyocytes were transfected with adenoviral pcDNA-H19 or empty vector and then exposed to high glucose (HG, 30 mmol/L) for 48 hours. The H19 expression was determined by real-time PCR. (**B**) Cardiomyocytes were stained with Annexin V/PI and then subjected to flow cytometry to detect apoptosis. (**C**) Cardiomyocytes were transfected with adenoviral H19 siRNA and/or adenoviral miR-675. The miR-675 expression was determined by real-time PCR. (**D**) The apoptotic rate of cardiomyocytes was measured by flow cytometry following Annexin V/PI staining. *P < 0.05.

High glucose was found to be associated with reduced H19 expression and elevated cardiomyocyte apoptosis (Fig. 4A,B). The expression of miR-675 was downregulated in cardiomyocytes transfected with H19 siRNA, which consequently resulted in increased apoptosis. However, enforced expression of miR-675 could suppress apoptosis in cardiomyocytes with H19 siRNA transfection, suggesting that H19 downregulation promotes apoptosis via inhibition of miR675 (Fig. 4C,D).

### Apoptosis-related gene VDAC1 is a direct target of miR-675

**Figure 5:**
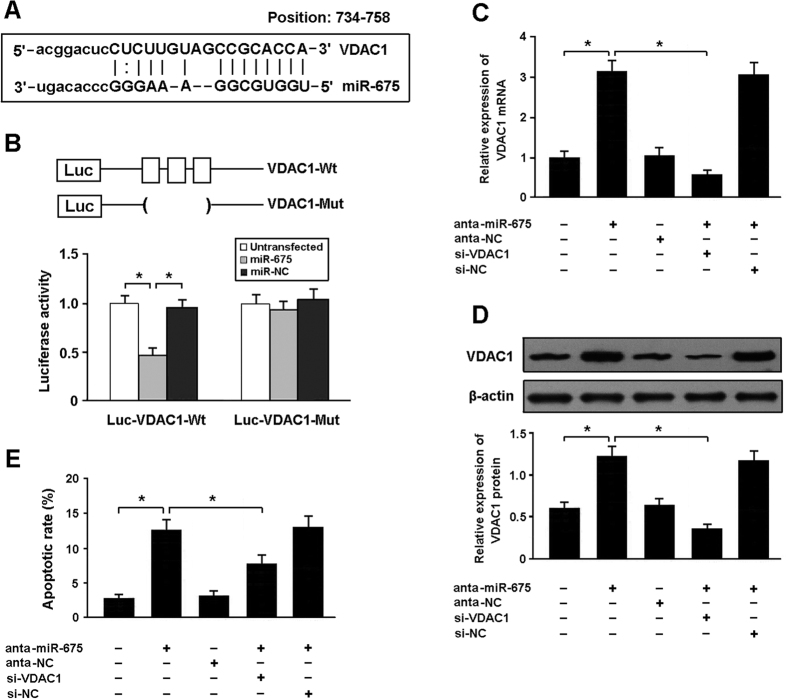
Apoptosis-related gene VDAC1 is a direct target of miR-675. (**A**) VDAC1 was predicted as a target gene of miR-675 using miRBase. (**B**) HEK293 cells were transfected with miR-675 mimic and luciferase constructs of VDAC1 3′UTR (Luc-VDAC1-Wt) or mutant (Luc-VDAC1-Mut). Luciferase activity was detected 48 hours after transfection. (**C**,**D**) Cardiomyocytes were transfected with miR-675 antagomir and/or adenoviral VDAC1 siRNA. The mRNA and protein expression of VDAC1 was determined by real-time PCR and Western blot. (**E**) The apoptotic rate of cardiomyocytes was detected by flow cytometry after Annexin V/PI staining. *P < 0.05.

Among the putative targets of miR-675, we focused on VDAC1, which belongs to the mitochondrial porin family and is involved in the modulation of apoptosis (Fig. 5A). The 3′-UTR of VDAC1 was fused to the luciferase coding region and transfected into HEK293 cells with miR-675 mimic. The luciferase assay indicated that VDAC1 was a direct target of miR-675 (Fig. 5B). The use of mutant derivatives in the miRNA recognition site confirmed the specificity of repressing activity. The expression of VDAC1 was upregulated in cardiomyocytes transfected with miR-675 antagomir, which consequently contributed to increased apoptosis. However, knockdown of VDAC1 could inhibit apoptosis in cardiomyocytes with miR-675 antagomir transfection, suggesting that miR-675 downregulation induces apoptosis by increasing VDAC1 expression (Fig. 5C–E).

### High glucose promotes apoptosis by regulating H19/VDAC1 pathway

**Figure 6:**
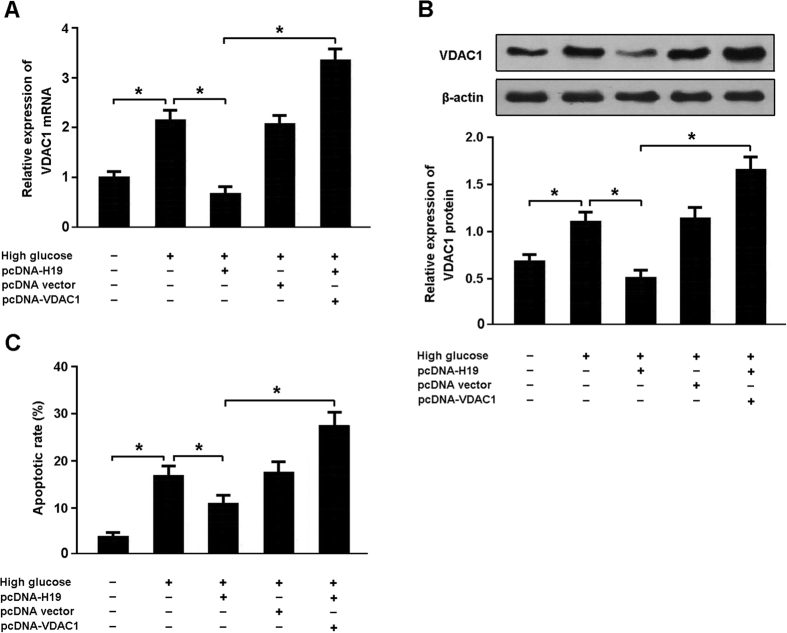
High glucose promotes apoptosis by regulating H19/VDAC1 pathway. (**A**,**B**) Cardiomyocytes were transfected with pcDNA-H19 and/or pcDNA-VDAC1 prior to exposure to high glucose (30 mmol/L) and the expression of VDAC1 was analyzed using real-time PCR and Western blot. (**C**) Cardiomyocytes were stained with Annexin V/PI and the apoptotic rate was determined by flow cytometry. *P < 0.05.

Cardiomyocytes were transfected with pcDNA-H19 and/or pcDNA-VDAC1 prior to exposure to high glucose and cellular apoptosis was determined by flow cytometry (Fig. 6A–C). The results indicated that high glucose was associated with increased VDAC1 expression and cardiomyocyte apoptosis, while enforced expression of H19 downregulated VDAC1 levels and inhibited apoptosis in cardiomyocytes exposed to high glucose. On the contrary, overexpression of both H19 and VDAC1 could promote apoptosis in high glucose-exposed cardiomyocytes.

## Discussion

DCM, an important cardiovascular complication in diabetic patients, carries a substantial risk for the subsequent development of heart failure and increased mortality. In the present study, we established a streptozocin-induced diabetic rat model to investigate the potential role of lncRNA H19 in the pathogenesis of DCM. Our results showed that H19 expression was significantly downregulated in the myocardium of diabetic rats, which might be associated with increased cardiomyocyte apoptosis and impaired cardiac function. We then performed *in vitro* experiments using cultured neonatal rat cardiomyocytes, and our findings revealed that high glucose could induce apoptosis by regulating H19/miR-675/VDAC1 pathway.

H19 is located on chromosome 11p15.5 and lies within 200 kbp downstream of the IGF-2 gene. Altered gene expression at the H19/IGF2 locus has been shown to be associated with malignancies and developmental disorders^11^,^12^. H19 can bind to and recruit the histone methyltransferase EZH2 at the E-cadherin promoter, leading to an increase in H3K27me3 repressive marks and to the silencing of the E-cadherin gene in bladder cancer^13^. The multifaceted function of H19 is also illustrated by its interaction with miRNAs. H19 can act as miRNA sponge to sequester miR-106a as well as the miR-let7 family members^14^,^15^. Moreover, H19 can serve as a precursor of miR-675 that will in turn, post-translationally regulate a number of target genes involved in cell proliferation and differentiation^16^,^17^,^18^.

miRNAs are a class of small noncoding RNAs that modulate the expression of target genes by binding to their 3′-UTR regions, leading to translational repression or mRNA degradation. In this study, we found that both miR-675 and its precursor H19 were downregulated in cardiomyocytes exposed to high glucose. Among the putative targets of miR-675, we focused on VDAC1, a gene encoding a voltage-dependent anion channel protein as a major component of the outer mitochondrial membrane. We cloned the 3′UTR of VDAC1 downstream of a luciferase reporter construct and cotransfected it into HEK293 cells with miR-675 mimic. The results of luciferase assay suggested that VDAC1 might be a direct target of miR-675.

Apoptosis, which is characterized by cell shrinkage, plasma membrane blebbing, chromatin compaction and nuclear fragmentation, plays critical roles in the pathogenesis of DCM^3^. During mitochondrial-mediated apoptosis, cytochrome c is released from mitochondrial into cytosol and subsequently activates caspase-9. The activated caspase-9 cleaves procaspase-3 to generate active caspase-3 which contributes to the cleavage of cellular target proteins^19^. Bax and Bcl-2 are two major Bcl-2 family members actively involved in the regulation of mitochondrial apoptotic pathway^20^. In the present study, our findings indicated that the cleaved caspase-3 expression and Bax/Bcl-2 ratio were significantly increased in the myocardium of diabetic rats and enforced expression of H19 could attenuate high glucose-induced apoptosis in cardiomyocytes.

VDAC1 belongs to the mitochondrial porin family and can facilitate the exchange of metabolites and ions across the outer mitochondrial membrane. VDAC1 is recognized as a key protein in the process of mitochondria-mediated apoptosis since it is the proposed target for the pro- and anti-apoptotic proteins of Bcl-2 family and due to its function in the release of apoptotic proteins located in the intermembranal space^21^,^22^. A previous study reported that injection of anti-VDAC1 antibodies into cells prevented Bax-induced cytochrome c release and the loss of mitochondrial membrane potential, which consequently resulted in decreased apoptosis^23^. In the present study, our findings demonstrated that the H19/miR-675 axis participated in the regulation of mitochondrial apoptotic pathway by targeting VDAC1.

In summary, these data revealed a novel function of H19/miR-675/VDAC1 pathway in the regulation of high glucose-mediated apoptosis, which may provide valuable insights for understanding the pathogenic role of lncRNA H19 in the development of DCM.

## Additional Information

**How to cite this article**: Li, X. *et al*. lncRNA H19/miR-675 axis regulates cardiomyocyte apoptosis by targeting VDAC1 in diabetic cardiomyopathy. *Sci. Rep.* **6**, 36340; doi: 10.1038/srep36340 (2016).

**Publisher’s note:** Springer Nature remains neutral with regard to jurisdictional claims in published maps and institutional affiliations.
